# Exosomes from Adipose Stem Cells Promote Diabetic Wound Healing through the eHSP90/LRP1/AKT Axis

**DOI:** 10.3390/cells11203229

**Published:** 2022-10-14

**Authors:** Sen Ren, Jing Chen, Jiahe Guo, Yutian Liu, Hewei Xiong, Boping Jing, Xiaofan Yang, Gongchi Li, Yu Kang, Cheng Wang, Xiang Xu, Zhenyu Liu, Maojie Zhang, Kaituo Xiang, Chengcheng Li, Qianyun Li, Hans-Günther Machens, Zhenbing Chen

**Affiliations:** 1 Department of Hand Surgery, Union Hospital, Tongji Medical College, Huazhong University of Science and Technology, No. 1277 Jiefang Avenue, Wuhan 430022, China; 2 Department of Neurosurgery, Zhongnan Hospital of Wuhan University, Wuhan 430071, China; 3 Department of Emergency Surgery, Union Hospital, Tongji Medical College, Huazhong University of Science and Technology, Wuhan 430022, China; 4 Department of Ultrasound, Union Hospital, Tongji Medical College, Huazhong University of Science and Technology, Wuhan 430022, China; 5 Department of Plastic and Hand Surgery, Technical University of Munich, D-80333 Munich, Germany

**Keywords:** adipose-derived stem cell, exosomes, diabetic wound, oxidative stress, heat shock protein 90

## Abstract

Oxidative damage is a critical cause of diabetic wounds. Exosomes from various stem cells could promote wound repair. Here, we investigated the potential mechanism by which exosomes from adipose-derived stem cells (ADSC-EXOs) promote diabetic wound healing through the modulation of oxidative stress. We found that ADSC-EXOs could promote proliferation, migration, and angiogenesis in keratinocytes, fibroblasts, and endothelial cells. Furthermore, ADSC-EXOs reduced the reactive oxygen species (ROS) levels in these cells and protected them against hypoxic and oxidative stress damage. Finally, the local injection of ADSC-EXOs at wound sites significantly increased collagen deposition and neovascularization while reducing ROS levels and cell death; thus, it led to accelerated diabetic wound closure. The mechanism underlying ADSC-EXO functions involved heat-shock protein 90 (HSP90) expressed on the cell surface; these functions could be inhibited by an anti-HSP90 antibody. Exosomal HSP90 could bind to the low-density lipoprotein receptor-related protein 1 (LRP1) receptor on the recipient cell membrane, leading to activation of the downstream AKT signaling pathway. Knockdown of LRP1 and inhibition of the AKT signaling pathway by LY294002 in fibroblasts was sufficient to impair the beneficial effect of ADSC-EXOs. In summary, ADSC-EXOs significantly accelerated diabetic wound closure through an exosomal HSP90/LRP1/AKT signaling pathway.

## 1. Introduction

Diabetic wounds are common complications of diabetes mellitus; such wounds pose a considerable clinical challenge [1]. Because delayed or non-healing wounds can lead to amputation and mortality, they constitute a heavy burden on patients’ families and society [2]. Currently, there are no effective treatment methods for such wounds. Oxidative stress damage, mediated by excessive production of reactive oxygen species, is a critical obstacle to healing in diabetic wounds [2,3]. Under diabetic conditions, local hypoxia and high glucose levels cause mitochondria to produce large amounts of ROS [4]. The presence of high ROS concentrations leads to sustained pro-inflammatory cytokine secretion and matrix metalloproteinase overproduction [5]. Furthermore, excessive ROS can impair skin cells (e.g., endothelial cells, keratinocytes, and fibroblasts), resulting in restricted neovascularization, decreased granulation tissue formation, and extracellular matrix deposition [6].

Previous studies have demonstrated that mesenchymal stem cells (MSCs) have therapeutic effects on diabetic wound healing [7]. However, the clinical implementation of MSC-based therapies has been limited by challenges such as immune rejection, risk of tumorigenesis, low transplantation efficacy, and ethical conflicts [8]. There is increasing evidence that the beneficial effects of MSCs mainly rely on their paracrine factors (e.g., growth factors, cytokines, and extracellular vesicles) [9]. Exosomes are small extracellular vesicles (30–150 nm in diameter) that participate in intercellular communication by delivering proteins, lipids, DNA, and RNA [10]. Recent studies have shown that exosomes from different MSCs can accelerate diabetic wound healing [11,12,13]. Adipose-derived mesenchymal stem cells (ADSCs) are regarded as highly promising stem cells resources because of their easy harvest, abundant content, and low immunogenicity characteristics [14]. Recent studies have also shown that ADSC-EXOs can promote skin wound healing through various mechanisms [15,16,17]. However, the specific effects of ADSC-EXOs on diabetic wound healing and the underlying mechanism require further investigation.

The heat-shock protein (HSP) family consists of a group of highly conserved proteins that respond to multiple stresses (e.g., heat, hypoxia, trauma, and starvation) [18]. Most HSPs act as molecular chaperones to assist protein folding and repair damaged proteins [19]. Initial investigations suggested that HSPs only functioned intracellularly. However, more recent studies have revealed that HSPs can be secreted into the external environment to mediate cellular communication; they have important roles in skin wound healing [20]. Defects in HSP function associated with diabetes might contribute to the delayed healing in diabetic wounds. Uncontrolled oxidative stress is a typical component of diabetes-related complications. Thus, there is a need to explore the antioxidant ability of HSPs for the management of diabetic wounds. Previous studies have shown that extracellular HSP90 (eHSP90) participates in normal wound healing; exogenous administration of eHSP90 could promote skin cell migration, thus accelerating the closure of normal and diabetic wounds [21,22,23]. The functions of extracellular HSPs depend on their interactions with cellular receptors such as Toll-like receptors 2 and 4 (TLR2 and TLR4) and low-density lipoprotein receptor-related protein 1 (LRP1) [24]. Because they lack signal peptides, HSPs are secreted into the extracellular environment mainly through exosomes [21,25]. Therefore, eHSP90 delivery through ADSC-EXOs could have therapeutic benefits.

In the present study, we hypothesized that ADSC-EXOs communicate with skin cells to provide signals that protect the skin cells against oxidative stress damage. We demonstrated that ADSC-EXOs could reduce intracellular ROS levels in skin cells while protecting skin cells from excessive ROS- and hypoxia-induced cell death both in vivo and in vitro. These protection and antioxidant effects were mediated by HSP90 present on the exosomal surface, which bound to LRP1 and activated the downstream AKT signaling pathway. Our findings provide important insights for therapeutic management of diabetic wound healing.

## 2. Materials and Methods

### 2.1. Cell Lines and Reagents

Human umbilical vein endothelial cells (HUVECs; #GDC166) and HaCaT cells (#GDC106) were purchased from the China Center of Type Culture Collection (CCTCC, Wuhan, China) and cultured in accordance with the supplier’s instructions. The inhibitors of PI3K (LY294002) and ERK1/2 (U0126) were purchased from MedChemExpress (Monmouth Junction, NJ, USA; HY-10108 and HY-12031A). Normal mouse IgG and anti-HSP90 mouse monoclonal IgG were purchased from Santa Cruz Biotechnology (Dallas, TX, USA). The following primary antibodies were utilized: anti-AKT (4691S), anti-p-AKT (4060S), anti-ERK (4695S), anti-p-ERK (4370S), anti-NF-κb (8242T), and anti-p-NF-κb (3033T) (all from Cell Signaling Technology, Danvers, MA, USA); anti-LRP1 (ab92544), anti-CD9 (ab236630), anti-CD63 (ab134045), and anti-GAPDH (ab8245) (all from Abcam, Waltham, MA, USA); anti-GM130 (11308) and anti-ꞵ-tubulin (66240) (both from Proteintech, Wuhan, China); and anti-HSP70 (sc-32239) and anti-HSP90 (sc-13119) (both from Santa Cruz, Dallas, TX, USA).

### 2.2. Culture and Characterization of Cells

Human adipose tissues and foreskins were obtained in accordance with procedures approved by the Ethics Committee at the Tongji Medical Collage of Huazhong University of Science and Technology. Primary fibroblasts were isolated from foreskins derived from routine pediatric circumcisions, using previously described protocols [26]. ADSCs were isolated and cultured in accordance with our established protocol [27]; ADSCs at passages 3–8 were used for experiments. Flow cytometry was conducted to identify ADSC phenotypes. ADSCs were incubated for 1 h with anti-CD34-BV421, anti-CD44-APC, anti-CD105-PE, anti-CD73-FITC, anti-CD31-FITC, and anti-CD90-FITC antibodies (all from Biolegend, San Diego, CA, USA); fluorescence was observed thereafter. For multi-lineage differentiation potential assays, ADSCs were incubated separately with adipogenic medium and osteogenic differentiation medium (Cyagen Biosciences Inc., Guangzhou, China). Cells were stained with Oil Red O and Alizarin Red, respectively. Subsequently, the stained cells were imaged using a microscope.

### 2.3. Isolation and Identification of ADSC-EXOs

ADSC-EXOs were isolated from the supernatant of adipose stem cells by differential centrifugation as described previously [28]. Briefly, the culture media were centrifuged at 1000× *g* for 10 min, 3000× *g* for 25 min, and 13,000× *g* for 40 min to eliminate dead cells, debris, and large macrovesicles separately. Then, the supernatants were centrifuged at 120,000× *g* for 70 min twice to obtain the exosomes pellets. Finally, the exosomes pellets were resuspended in phosphate-buffered saline (PBS) and stored at −80 °C. The protein concentration was determined by a BCA protein assay kit (Beyotime Biotechnology, Shanghai, China). The morphology of ADSC-EXOs was captured by transmission electron microscope (Hitachi, Tokyo, Japan). The size and concentration of the ADSC-EXOs was evaluated by nanoparticle tracking analysis (Wayen Biotechnologies, Shanghai, China). The exosomal-positive markers CD63, CD9, HSP70, and -negative markers GM130 and β-tubulin were detected by Western blot.

### 2.4. Intracellular Uptake of ADSC-EXOs

ADSC-EXOs were labeled with PKH26 fluorescent dye (Sigma-Aldrich, St. Louis, MO, USA) in accordance with the manufacturer’s instructions. Cells were seeded in confocal dishes and incubated with PKH26-labeled ADSC-EXOs for various intervals. Phalloidin (Yeasen Biotech Co., Shanghai, China) and DAPI (Solarbio, Beijing, China) were used to label the cytoskeleton and nucleus before image acquisition. Images were acquired using a confocal microscope (Nikon, Tokyo, Japan).

### 2.5. Proliferation, Migration, and Tube Formation Assays

Fibroblasts, HUVECs, and HaCaT cells were treated with phosphate-buffered saline (PBS) or ADSC-EXOs (20 µg/mL) for 48 h; cell proliferation was evaluated by EdU kits (Beyotime Biotechnology). The migration abilities of fibroblasts, HUVECs, and HaCaT cells were assayed using 24-well Transwell Chambers (Corning Inc., Corning, NY, USA). In brief, cells suspended in Dulbecco’s modified Eagle medium without serum were added to the upper chamber and treated with PBS or ADSC-EXOs (5 µg/mL and 10 µg/mL). The lower chamber was filled with 600 µL of complete culture media consisting of 10% fetal bovine serum. After incubation at 37 °C for 24 h, cells that had migrated to the bottom surface were stained with crystal violet (Solarbio, Beijing, China). For the tube formation assay, HUVECs were seeded in 48-well plates that had been precoated with Matrigel Basement Membrane Matrix (BD Biosciences, NJ, USA). After incubation with PBS and ADSC-EXOs (10 µg/mL or 20 µg/mL) for 2 h, 4 h, and 8 h, tube formation was imaged by microscopy. The total number of loops, nodes, and branch points was calculated and used to quantify the tubular networks.

### 2.6. In Vitro Model of Hypoxia and Oxidative Stress

Cellular models of oxidative stress and hypoxia were generated using H_2_O_2_ and cobalt chloride (CoCl_2_) (both from Sigma-Aldrich, St. Louis, MO, USA). HaCaT cells, fibroblasts, and HUVECs were seeded and treated with different concentrations of H_2_O_2_ for 6 h or CoCl_2_ for 24 h. Cell viabilities were analyzed using a CCK-8 kit (Dojindo, Kumamoto, Japan). The absorbance values at 450 nm were measured using a microplate reader (Tecan, Männedorf, Switzerland). Cell viabilities were also assessed by Calcein-AM/PI double staining (Yeasen Biotech Co., Shanghai China). Subsequently, the stained cells were imaged using a fluorescence microscope (Olympus, Tokyo, Japan).

### 2.7. Intracellular ROS Detection

Intracellular ROS levels in the skin cells of in vitro models were analyzed using a ROS detection kit (2,7-dichlorofluorescein diacetate, DCFH-DA) from the Nanjing Jiancheng Bioengineering Institute (Nanjing, China). After different treatments, cells were incubated with DCFH-DA for 30 min, in accordance with the manufacturer’s instructions. Then, cellular fluorescence was observed using a fluorescence microscope or a flow cytometer (BD Biosciences, NJ, USA).

### 2.8. Apoptosis Assay

After different treatments, fibroblasts were incubated with a Annexin V-FITC Apoptosis Detection Kit (Sigma-Aldrich, St. Louis, MO, USA), in accordance with the manufacturer’s instructions. The fluorescence of stained cells was detected by fluorescence-activated cell sorting using a flow cytometer (BD Biosciences, NJ, USA) and analyzed by FlowJo V10 software.

### 2.9. Proteomic Analysis of ADSC-EXOs

Equal exosomal proteins (20 µg) were separated on sodium dodecyl sulfate-polyacrylamide gel (SDS-PAGE) and subjected to silver staining (Beyotime Biotechnology, Shanghai, China). Then, the gels were cut into seven parts according to the protein molecular weight. Next, proteins in the stained bands were identified with the LC-MS/MS experiment (maXis impact UHR-TOF, Bruker, Germany). Raw data files were possessed by the Data Analysis software (Compass data analysis v4.1, Bruker, Germany). Protein identification was performed utilizing PEAKS 8.5 software (BSI, Canada). The GO categories (http://geneontology.org) were used for defining the cellular component, molecular function, and biological process of involved proteins.

### 2.10. eHSP90 Function-Blocking Assay

IgG, anti-HSP90 antibody, and an equal volume of PBS were, respectively, mixed with ADSC-EXOs at a mass ratio of 10:1 and incubated at 37 °C for 30 min. The following are the three treatment groups used for subsequent experiments: EXOs groups, EXOs + IgG groups, and EXOs + anti-HSP90 groups.

### 2.11. Cell Transfection

For gene knockdown, sh-LRP1 and control lentiviruses were synthesized by Genechem Co., Ltd. (Shanghai, China). Cells were seeded into six-well plates for 1 day. The culture medium was then refreshed; cells were transfected with sh-LRP1 or control lentiviruses for 3 days. Gene-silencing efficiency was validated by Western blotting. The shRNA sequences are shown in Supplementary Table S1.

### 2.12. Western Blotting

Equal amounts of total protein (20–40 μg) were separated by sodium dodecyl sulfate-polyacrylamide gel electrophoresis and transferred onto polyvinylidene fluoride membranes. The membranes were blocked with 5% *w*/*v* bovine serum albumin for 1 h at room temperature, then incubated with primary antibodies overnight at 4 °C. Subsequently, membranes were incubated with secondary antibodies for 2 h and exposed to X-ray film for visualization by the BioSpectrum Imaging System (UVP, CA, USA).

### 2.13. Diabetic Wound Model

All animal experiments were approved by the Animal Care Committee of Tongji Medical College. The effects of ADSC-EXOs on diabetic wound healing were evaluated in a diabetic mouse model with a cutaneous wound. Male C57BL/6 mice (20–25 g) were used in the present study. Diabetes was induced by intraperitoneal injection of streptozotocin (50 mg/kg) for 5 consecutive days; control mice received an equal volume of citrate buffer. Blood glucose was monitored for 2 weeks thereafter. Mice with blood glucose levels > 16.7 mM (300 mg/dL) were regarded as diabetic mice; they were monitored for an additional 4 weeks before the induction of full-thickness skin wounds.

Mice were anesthetized by intraperitoneal injection of pentobarbital solution (50 mg/kg; Sigma-Aldrich). A full-thickness round skin wound (9 mm in diameter) was created on the back of each mouse; wound edges were sutured with silicone rings (1.0 cm in diameter) to prevent wound contraction. Then, the wounded mice were randomly divided into five treatment groups: Nor + PBS (normal mice treated with PBS); Dia + PBS (diabetic mice treated with PBS); Dia + EXOs (diabetic mice treated with EXOs); Dia + EXOs + IgG (diabetic mice treated with EXOs and IgG); and Dia + EXOs + anti-HSP90 (diabetic mice treated with EXOs and anti-HSP90 antibody). ADSC-EXOs (50 µg per mouse) and PBS (equal volume to the ADSC-EXOs suspension) were subcutaneously injected into five sites around the wounds once after the wounds were created. At days 0, 3, 8, and 13, the wounds were photographed and analyzed using ImageJ software (v1.46r).

### 2.14. Histological and Immunofluorescence Analysis

Wound tissue samples were harvested on day 13 after surgery and fixed using 4% paraformaldehyde. After dehydration by ethanol, the tissues were embedded in paraffin and sliced into 5 μm thick longitudinal sections. The degree of wound re-epithelialization was analyzed using hematoxylin and eosin staining. The degree of collagen deposition was evaluated using Masson’s staining. To evaluate wound angiogenesis, immunofluorescence staining of α-SMA and CD31 was performed in wound tissue samples. To evaluate cell proliferation in wound tissue, we performed immunofluorescence staining of Ki67 and immunohistochemical staining of proliferating cell nuclear antigen (PCNA). Cell apoptosis in tissues was determined using a TUNEL fluorescence kit (BD Biosciences, NJ, USA). To detect ROS levels in wound sections, frozen wound tissue samples were cryosectioned; they were then stained with dihydroethidium at room temperature for 1 h. Images were captured with a fluorescence microscope and analyzed using ImageJ software (v1.46r).

### 2.15. Statistics

All statistical data are shown as means ± standard errors of the mean; the data were evaluated using GraphPad Prism 7. Student’s *t*-test was applied to compare differences between two groups; one-way or two-way analysis of variance and Bonferroni post hoc analysis were used for comparisons of ≥3 groups. *p*-values < 0.05 were considered statistically significant (* *p* < 0.05, ** *p* < 0.01, *** *p* < 0.001, **** *p* < 0.0001).

## 3. Results

### 3.1. Characterization of ADSCs and ADSC-EXOs

Primary ADSCs were successfully extracted from human adipose tissue and identified by their morphology, surface markers, and multi-lineage differentiation potential. Flow cytometry analysis showed that large proportions of ADSCs expressed CD73 (98.4%), CD90 (94.5%), CD44 (99.6%), and CD105 (99.2%); small proportions of ADSCs expressed CD34 (1.42%) and CD31 (1.24%) (Figure S1A). Microscopy analysis revealed that ADSCs had typical fibroblast-like morphology and grew in a swirled pattern (Figure S1B). The presence of intracellular lipid droplets and calcium nodules indicated that ADSCs could differentiate into adipocytes and osteoblasts (Figure S1C,D). Exosomes were isolated from ADSC culture supernatants by differential ultracentrifugation. Transmission electron microscopy analysis showed that ADSC-EXOs had cup-shaped morphology (Figure S2A,B). Western blotting analysis indicated that ADSC-EXOs expressed exosomal markers CD63, CD9, and HSP70; they did not express GM103 or β-tubulin (Figure S2C). Nanoparticle tracking analysis revealed that the mean diameter of ADSC-EXOs was 126.3 ± 0.1 nm (Figure S2D). These results confirmed that ADSC-EXOs had successfully been isolated.

### 3.2. ADSC-EXOs Promote Skin Cell Proliferation, Migration, and Angiogenesis In Vitro

**Figure 1:**
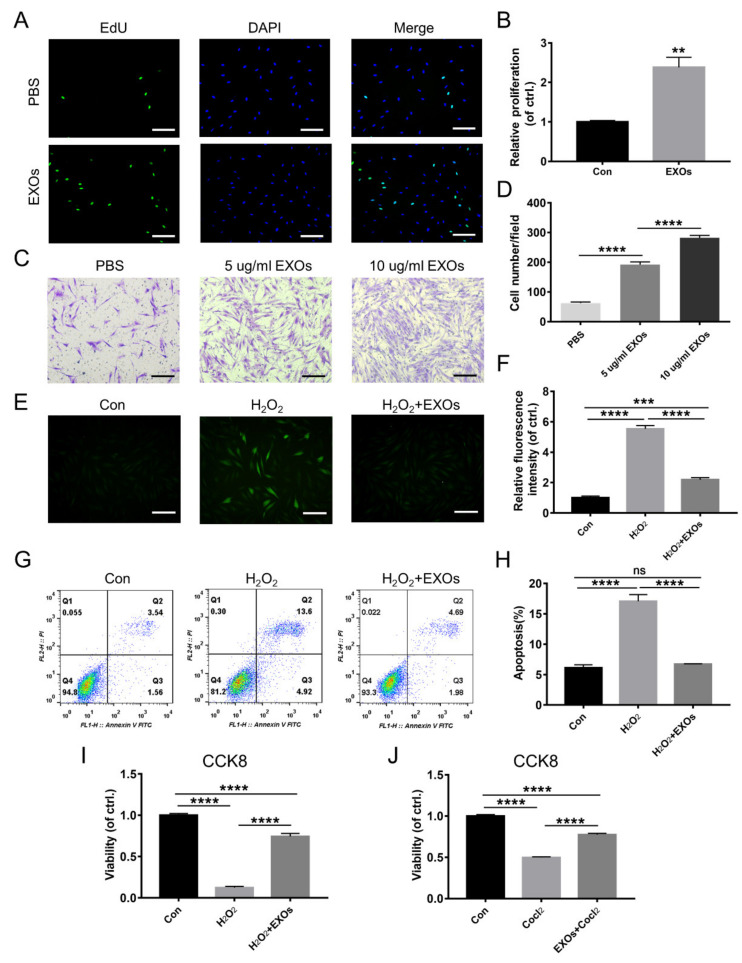
ADSC-EXOs promoted fibroblasts proliferation and migration and attenuated intracellular ROS levels and cell damages caused by hypoxia and oxidative stress. (**A**) Representative fluorescent images of proliferative fibroblasts after ADSC-EXOs treatment. (**B**) Quantitative analysis of the data in (**A**). (**C**) Representative images of migratory fibroblasts given ADSC-EXOs treatments. (**D**) Quantitative analysis of the data in (**C**). Fibroblasts were pre-treated with ADSC-EXOs or PBS for 24 h, then exposed under H_2_O_2_ for 6 h or CoCl_2_ for 24 h, and finally assessed by the following assays. (**E**) Representative images of DCFH fluorescence in fibroblasts given the above treatments. (**F**) Quantitative analysis of the relative DCFH fluorescence intensity. (**G**) Flow cytometry analysis of the cell apoptosis in each group. (**H**) Quantitative analysis of the apoptosis rate. (**I**) Quantitative analysis of the cell viability of fibroblasts under H_2_O_2_ exposure by CCK8 assay. (**J**) Quantitative analysis of the cell viability of fibroblasts under CoCl_2_ exposure by CCK8 assay. Scale bar: 100 μm. *n* = 3. ** *p* < 0.01, *** *p* < 0.001, **** *p* < 0.0001.

New granulation tissue formation requires precise interactions among keratinocytes, fibroblasts, and endothelial cells [29]. First, we investigated the effects of ADSC-EXOs on proliferation in these cells (i.e., keratinocytes, fibroblasts, and endothelial cells) by EdU assays. The percentages of proliferating cells were significantly increased after ADSC-EXO treatment; fibroblasts demonstrated the greatest benefit (Figure 1A,B and Figure S3). Next, the effects of ADSC-EXOs on the migration of HaCaT cells, fibroblasts, and HUVECs were evaluated by Transwell assays. As expected, after treatment with 5 µg/mL and 10 µg/mL ADSC-EXOs, the numbers of migrated HUVECs increased by 1.65-fold and 3.65-fold, respectively, compared with untreated controls; the corresponding numbers of migrated fibroblasts increased by 3.08-fold and 4.52-fold; and the corresponding numbers of migrated HaCaT cells increased by 1.71-fold and 3.11-fold (Figure 1C,D and Figure S4). Tube formation assays were used to evaluate the ability of ADSC-EXOs to promote angiogenesis in HUVECs. The numbers of capillary-like tubular structures and junctions were significantly increased after 2 h, 4 h, and 8 h of incubation with ADSC-EXOs (Figure S5). Taken together, these data demonstrated that ADSC-EXOs could enhance proliferation, migration, and angiogenesis in skin cells during in vitro experiments.

### 3.3. ADSC-EXOs Protect Skin Cells against Damage from Hypoxia and Oxidative Stress

We used H_2_O_2_ and CoCl_2_ to simulate an excess oxidative stress and hypoxia microenvironment in diabetic wounds. Flow cytometry analysis showed that H_2_O_2_ triggered a sharp increase in the intracellular ROS levels of HaCaT cells, fibroblasts, and HUVECs; this trend could be partially reversed by treatment with ADSC-EXOs (Figure S6A–F). DCFH fluorescence analysis also demonstrated that ADSC-EXO treatment could significantly attenuate H_2_O_2_-induced ROS overproduction in fibroblasts (Figure 1E,F). Furthermore, the proportion of apoptotic fibroblasts was increased after H_2_O_2_ exposure; ADSC-EXO treatment fully abolished this harmful effect, as demonstrated by flow cytometry analysis of Annexin V/PI (Figure 1G,H). CCK8 assays also revealed that ADSC-EXO treatment could partially protect skin cells against H_2_O_2_- and CoCl_2_-induced cell death; fewer than 65% of HUVECs survived after H_2_O_2_ or CoCl_2_ exposure, while ADSC-EXO treatment greatly increased the proportions of surviving HUVECs regardless of exposure (Figure S6G,H). The rate of HaCaT cell survival in the H_2_O_2_ model was increased from 73.8% to 96.0% by ADSC-EXO treatment; it was increased from 66.1% to 86.3% in the CoCl_2_ model (Figure S6I,J). Furthermore, the rate of fibroblast survival in the H_2_O_2_ model was increased from 12.5% to 74.4% by ADSC-EXO treatment; it was increased from 49.7% to 77.6% in the CoCl_2_ model (Figure 1I,J). In brief, ADSC-EXO treatment could reduce the intracellular ROS level in skin cells and protect those cells against damage from hypoxia and oxidative stress.

### 3.4. Internalization and Proteomic Analysis of ADSC-EXOs

**Figure 2:**
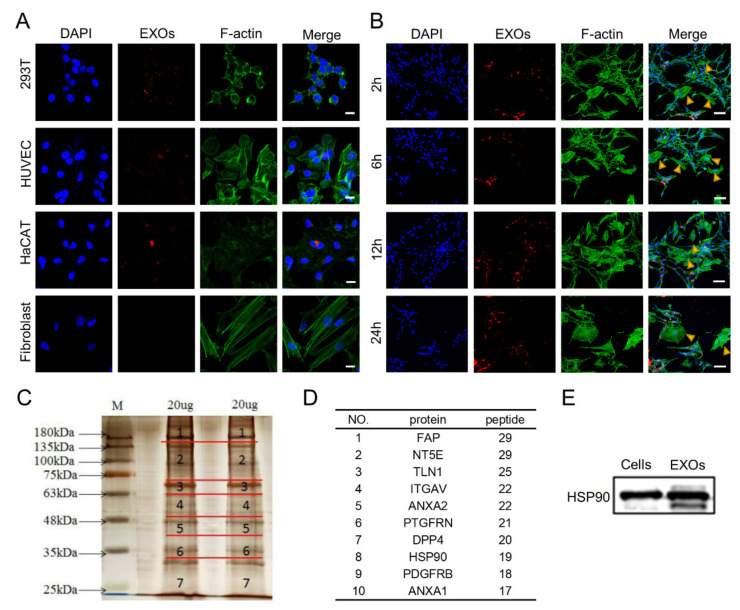
Internalization and protein analysis of ADSC-EXOs. (**A**) Confocal images of 293T, HaCAT, HUVECs, and fibroblasts incubated with 20 μg PKH26-labeled ADSC-EXOs for 24 h; scale bar: 20 μm. (**B**) Confocal images of co-cultured Schwann cells and fibroblasts incubated with PKH26-labeled ADSC-EXOs at different times; scale bar: 100 μm. (**C**) Silver staining analysis of ADSC-EXOs protein gels. (**D**) Illustration of the top ten high-abundance surface proteins in ADSC-EXOs. (**E**) Western blot analysis of HSP90 levels in ADSCs and ADSC-EXOs.

To characterize the fates of exosomes among recipient cells, we explored whether ADSC-EXOs could be internalized by skin cells. For this purpose, we labeled ADSC-EXOs with PKH26 and co-cultured them with 293T cells, HUVECs, HaCaT cells, or fibroblasts. Red fluorescence was clearly observed in the cytoplasm of 293T cells, HUVECs, and HaCaT cells; it was nearly absent from fibroblasts (Figure 2A). To confirm the reliability of the findings, fibroblasts were co-cultured with Schwann cells, which have been shown to internalize exosomes [28]; the co-cultured cells were then incubated with PKH26-labeled ADSC-EXOs for 2 h, 6 h, 12 h, or 24 h. Similarly, red fluorescence was observed in the cytoplasm of Schwann cells but was generally absent from fibroblasts (Figure 2B). We concluded that ADSC-EXOs were not easily internalized by fibroblasts. Thus, we presumed that the effect of ADSC-EXOs on fibroblasts mainly relied on ligand–receptor-induced intracellular signaling activation. This type of mechanism may also participate in the effects of ADSC-EXOs on keratinocytes and endothelial cells.

To investigate the exosomal membrane proteins that mediate the effects of ADSC-EXOs, we used mass spectrometry to identify the protein profiles of ADSC-EXOs (Figure 2C). In total, 1106 proteins were identified; gene ontology analysis was conducted to classify functional categories of all identified proteins (Tables S2 and S3). Gene ontology analysis showed that 77 proteins were located in the cell surface category; these were presumed to localize at the exosomal surface. Next, the 10 most abundant surface proteins were identified; these included HSP90 (Figure 2D). Previous studies have shown that HSP90 is present on the surface of tumor cell-secreted exosomes and has important roles in tumor invasion and metastasis [24]. Western blotting analysis also confirmed the high abundance of HSP90 in ADSC-EXOs (Figure 2E). Taken together, these data suggested that HSP90 might be the main effector of ADSC-EXOs.

### 3.5. eHSP90 Is Required for the Protective Effect of ADSC-EXOs

**Figure 3:**
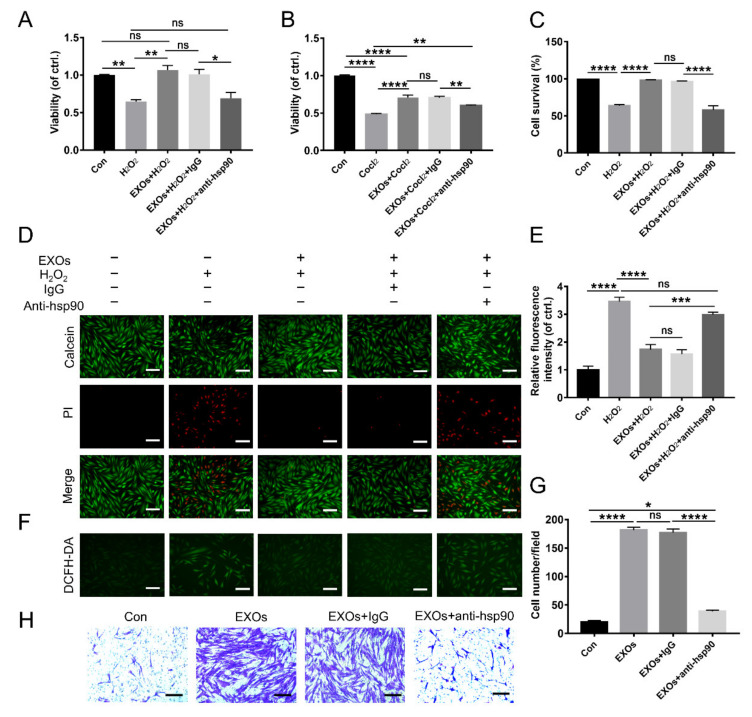
An anti-HSP90 antibody antagonized the beneficial effect of ADSC-EXOs. ADSC-EXOs were pre-incubated with PBS, IgG, and an anti-HSP90 antibody for 30 min at 37 °C and then used in the following assays. (**A**) CCK8 analysis of the cell viability of fibroblasts under H_2_O_2_ exposure in each group. (**B**) CCK8 analysis of the cell viability of fibroblasts under CoCl_2_ exposure in each group. (**C**) Quantitative analysis of the data in (**D**). (**D**) Evaluation of the cell viability by Calcein-AM/PI staining; the green color represents survival cells, and the red color represents dead cells. (**E**) Quantitative analysis of the relative fluorescence intensity in (**F**). (**F**) Representative DCFH fluorescent images of fibroblasts in each group. (**G**) Quantitative analysis of the data in (**H**). (**H**) Representative micrographs of migratory fibroblasts in each group. *n* = 5. Scale bar: 100 μm. ns *p* > 0.05, * *p* < 0.05, ** *p* < 0.01, *** *p* < 0.001, **** *p* < 0.0001.

To determine whether eHSP90 is essential for ADSC-EXO function, we used an anti-HSP90 antibody to neutralize this exosomal surface protein. CCK8 assays showed that ADSC-EXO treatment could significantly decrease H_2_O_2_- and CoCl_2_-induced cell death; this protective effect was eliminated upon pretreatment with an anti-HSP90 antibody rather than an IgG-isotype control (Figure 3A,B). This result was confirmed by Calcein-AM/PI staining analysis (Figure 3C,D). Additionally, H_2_O_2_ exposure caused a 3.46-fold increase in the intracellular ROS level in fibroblasts compared with untreated control fibroblasts; the intracellular ROS level was decreased (1.73-fold lower than untreated control fibroblasts) by ADSC-EXO treatment but increased again (2.99-fold higher than untreated control fibroblasts) upon pretreatment with an anti-HSP90 antibody (Figure 3E,F). These results suggested that the anti-HSP90 antibody could abolish the ability of ADSC-EXOs to protect fibroblasts from oxidative stress damage; they were also compatible with the findings in our Transwell assays to characterize the promigratory effect of exosomal surface HSP90. In the Transwell assays, we found that the numbers of migrated fibroblasts increased by 9.08-fold and 8.85-fold after treatment with EXOs or EXOs + IgG; these large increases were significantly suppressed by pretreatment of EXOs with an anti-HSP90 antibody (Figure 3G,H). However, the number of migrated fibroblasts remained 1.94-fold higher in the EXOs + anti-HSP90 group than in the control group. These data confirmed that the anti-HSP90 antibody could antagonize the promigratory effect of ADSC-EXOs. In conclusion, eHSP90 contributed to the effects of ADSC-EXOs on cell migration and protection.

### 3.6. eHSP90 Activates the Downstream AKT Signaling Pathway

**Figure 4:**
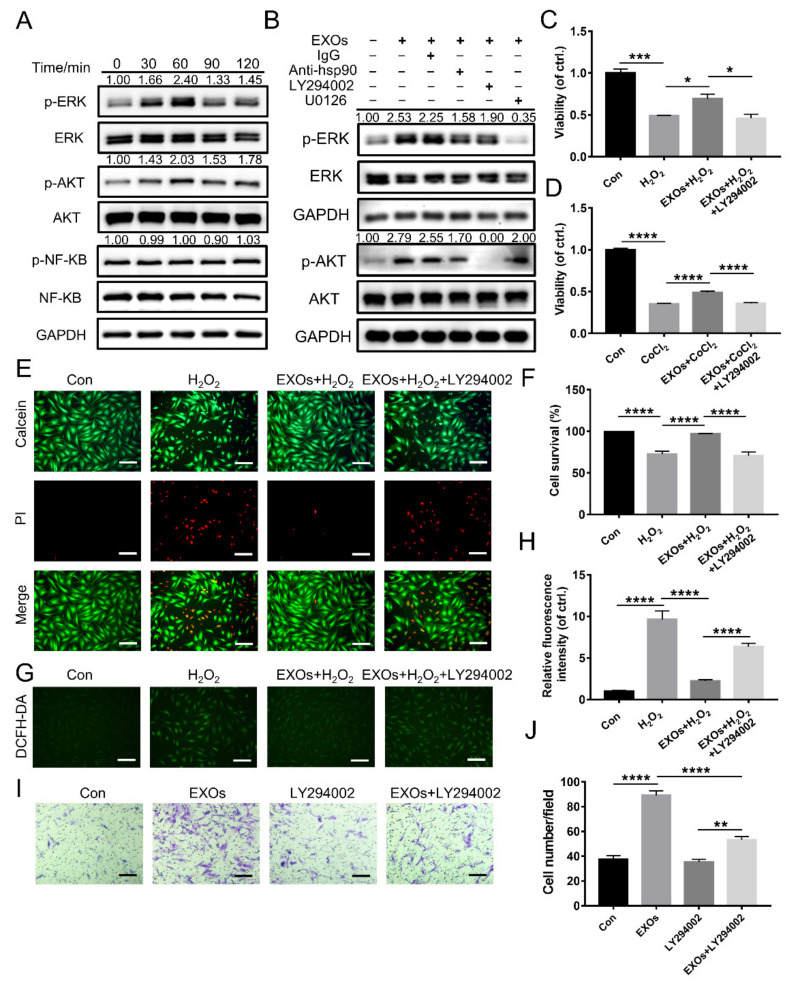
The effect of ADSC-EXOs relied on the activation of AKT signaling pathway. (**A**) Western blot analysis of the activation of AKT, ERK, and NF-κB pathways in fibroblasts after treated with ADSC-EXOs at different times (*n* = 3). (**B**) Representative blots showing the activation of AKT and ERK pathways in fibroblasts following various treatments (*n* = 3). Fibroblasts were pre-treated with EXOs, EXOs + LY294002, or PBS for 24 h; then exposed under H_2_O_2_ for 6 h or CoCl_2_ for 24 h; and finally assessed by the following assays. (**C**) CCK8 analysis of the cell viability of fibroblasts under H_2_O_2_ exposure in each group. (**D**) CCK8 analysis of the cell viability of fibroblasts under CoCl_2_ exposure in each group. (**E**) Evaluation of the fibroblasts viability by Calcein-AM/PI staining. (**F**) Quantitative analysis of the data in (**E**). (**G**) Representative DCFH fluorescent images of fibroblasts in each group. (**H**) Quantitative analysis of the relative fluorescence intensity in (**G**). (**I**) Representative micrographs of migratory fibroblasts given above treatments. (**J**) Quantitative analysis of the data in (**I**). *n* = 5. Scale bar: 100 μm. * *p* < 0.05, ** *p* < 0.01, *** *p* < 0.001, **** *p* < 0.0001.

Western blotting analysis was conducted for further exploration of the intracellular signaling pathways activated in fibroblasts during incubation with ADSC-EXOs. We focused on ERK, AKT, and NF-κB pathways because they are reportedly activated by eHSP90 [22,30]. As shown in Figure 4A, ADSC-EXO treatment induced time-dependent phosphorylation of both AKT (p-AKT) and ERK (p-ERK); the maximum increases in p-AKT and p-ERK levels (2.40-fold and 2.03-fold, respectively) were achieved at 60 min after ADSC-EXO treatment; however, there was no increase in the phosphorylation of NF-κB (p-NF-κB). Furthermore, ADSC-EXO treatment also induced time-dependent phosphorylation of both AKT (p-AKT) and ERK (p-ERK) in HUVEC and HaCaT (Figure S7). Next, to clarify whether AKT and ERK signaling pathways were involved in the protective effects of ADSC-EXOs, fibroblasts were pre-treated with LY294002 (AKT inhibitor) and U0126 (ERK inhibitor). First, we confirmed that the ADSC-EXO-induced phosphorylation of AKT and ERK could be entirely inhibited by LY294002 and U0126, respectively; each type of phosphorylation could also be suppressed by the use of anti-HSP90 antibody (Figure 4B). Next, we used CCK8 assays to investigate the protective effects of ADSC-EXOs on fibroblasts exposed to H_2_O_2_ and CoCl_2_; these protective effects could be totally abolished by LY294002 (Figure 4C,D). This result was confirmed by Calcein-AM/PI staining analysis (Figure 4E,F). Similarly, DCFH fluorescence analysis demonstrated that LY294002 could attenuate the ability of ADSC-EXOs to reduce the intracellular ROS level (Figure 4G,H). Notably, U0126 could not antagonize the protective effect of ADSC-EXOs; it greatly promoted fibroblast survival under oxidative stress damage (Figure S8A). These data indicated that eHSP90 protected fibroblasts against hypoxic and oxidative stress damage through activation of the AKT signaling pathway.

Transwell migration assays showed that the effect of ADSC-EXOs on promoting fibroblasts migration was significantly suppressed by LY294002; however, LY294002 alone had no effect on cell migration (Figure 4I,J). In contrast, U0126 alone promoted fibroblast migration but did not affect the promigratory function of EXOs (Figure S8B,C). These data confirmed that ADSC-EXOs promoted cell migration by activating the AKT signaling pathway rather than the ERK signaling pathway. Thus, we concluded that eHSP90 promoted cell migration and survival through activation of the AKT signaling pathway.

### 3.7. eHSP90 Interacts with LRP1 on Fibroblasts

**Figure 5:**
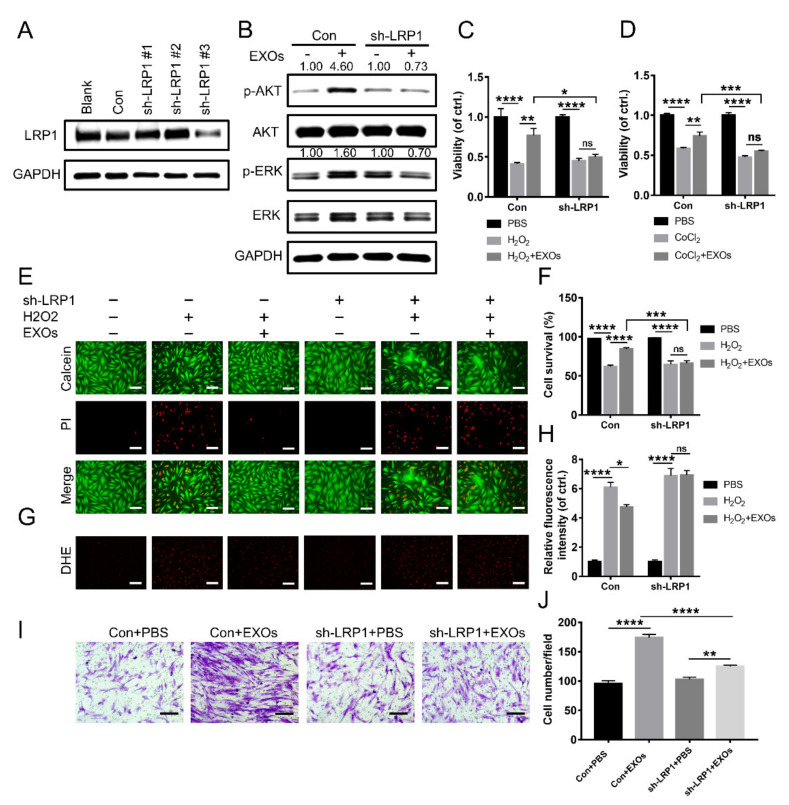
ADSC-EXOs regulated cell function via binding to the receptor LRP1 on fibroblasts. (**A**) Western blot analysis of the gene-silencing efficiency of sh-LRP1 lentivirus vectors (*n* = 3). (**B**) Western blot analysis of the activation of AKT and ERK pathways in control and LRP1 knockdown fibroblasts after the EXOs treatment (*n* = 3). Control and LRP1 knockdown fibroblasts were pre-treated with ADSC-EXOs or PBS for 24 h, then exposed under H_2_O_2_ for 6 h or CoCl_2_ for 24 h, and finally assessed by the following assays. (**C**) CCK8 analysis of the cell viability of fibroblasts under H_2_O_2_ exposure. (**D**) CCK8 analysis of the cell viability of fibroblasts under CoCl_2_ exposure. (**E**) Evaluation of the fibroblasts viability by Calcein-AM/PI staining. (**F**) Quantitative analysis of the data in (**E**). (**G**) Representative DHE fluorescent images of fibroblasts in each group. (**H**) Quantitative analysis of the relative fluorescence intensity in (**G**). (**I**) Representative micrographs of migratory fibroblasts after the EXOs treatment. (**J**) Quantitative analysis of the data in (**I**). *n* = 5. Scale bar: 100 μm. * *p* < 0.05, ** *p* < 0.01, *** *p* < 0.001, **** *p* < 0.0001.

A previous study demonstrated that eHSP90 binding to LRP1 could promote skin cell migration [22]. To further investigate whether ADSC-EXO function was mediated by LRP1, we designed three pairs of shRNAs to inhibit LRP1 expression in fibroblasts. Of these pairs, sh-LRP1#3 showed the highest interference efficiency; it was used in subsequent experiments (Figure 5A). Western blotting analysis showed that ADSC-EXOs significantly increased the levels of p-AKT and p-ERK in control fibroblasts but not in LRP1 knockdown fibroblasts (Figure 5B). As expected, ADSC-EXOs exhibited a pro-survival effect against H_2_O_2_ and CoCl_2_ exposures in control fibroblasts; this beneficial effect was not observed in LRP1 knockdown fibroblasts (Figure 5C,D). This result was confirmed by Calcein-AM/PI staining analysis (Figure 5E,F). Dihydroethidium fluorescence analysis also demonstrated that ADSC-EXOs could partially reverse the H_2_O_2_-induced enhancement of intracellular ROS level in control fibroblasts but not in LRP1 knockdown fibroblasts (Figure 5G,H). Furthermore, the number of migrated cells was greatly increased by EXO treatment in control fibroblasts but only slightly increased in LRP1 knockdown fibroblasts (Figure 5I,J). These results demonstrated that the promigratory and protective effects of ADSC-EXOs were related to the presence of LRP1 on the recipient cell surface. Therefore, ADSC-EXOs activated the AKT signaling pathway in a LRP1-dependent manner.

### 3.8. ADSC-EXOs Promote Diabetic Wound Closure via eHSP90

**Figure 6:**
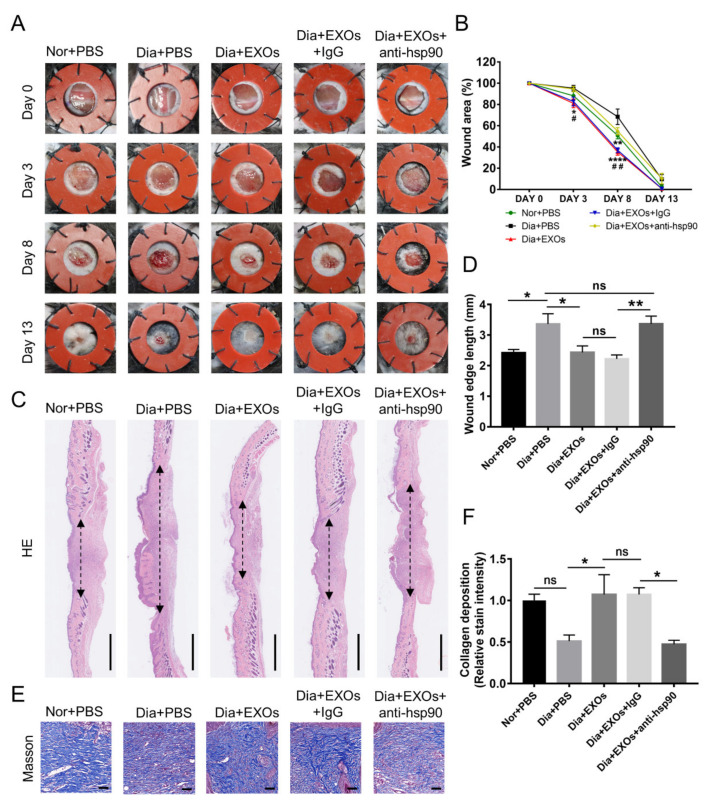
ADSC-EXOs accelerated skin wound healing in diabetic mice via eHSP90. (**A**) Representative images of skin wounds at days 0, 3, 8, and 13. (**B**) Quantitative analysis of the wound-closure rates in different treatment groups; * vs. Dia + PBS, ^#^ vs. Dia + EXOs + anti-hsp90. (**C**) H & E staining analysis of wound sections on day 13 post wounding; the double-headed arrows represent edges of the granulation; scale bar: 1 mm. (**D**) Quantification of the wound edge length shown in (**C**). (**E**) Evaluation of collagen deposition by Masson’s trichrome staining in each group; scale bar: 50 μm. (**F**) Qualification of the stain intensity of blue collagen shown in (**E**). *n* = 7. * *p* < 0.05, ** *p* < 0.01, **** *p* < 0.0001, ## *p* < 0.01.

To determine whether ADSC-EXOs could accelerate diabetic wound healing through interactions with surface protein HSP90, we established a full-thickness diabetic wound model. The wound-closure rate was more rapid in diabetic mice treated with EXOs than in untreated mice at day 8 after wounding; this EXO-induced rapid healing was significantly suppressed by an anti-HSP90 antibody (Figure 6A,B). Furthermore, wound healing was substantially slower in diabetic mice than in healthy mice. Next, we performed a histological analysis to assess the degrees of wound healing and regeneration. Hematoxylin and eosin staining analysis revealed that wound edges were significantly narrowed in the EXO-treated groups; anti-HSP90 antibody could completely inhibit this effect (Figure 6C,D). Furthermore, Masson’s trichrome staining analysis showed that collagen deposition was more extensive and better-organized in wounds treated with EXOs; this benefit was not observed in wounds treated with EXOs that had been incubated with an anti-HSP90 antibody (Figure 6E,F). Strikingly, ADSC-EXO treatment caused the levels of collagen deposition and wound edge contraction in diabetic mice to approach the levels found in healthy mice. These data suggested that ADSC-EXOs could promote collagen deposition and accelerate wound healing via eHSP90.

### 3.9. ADSC-EXOs Promote ROS Scavenging and Angiogenesis via Surface HSP90

**Figure 7:**
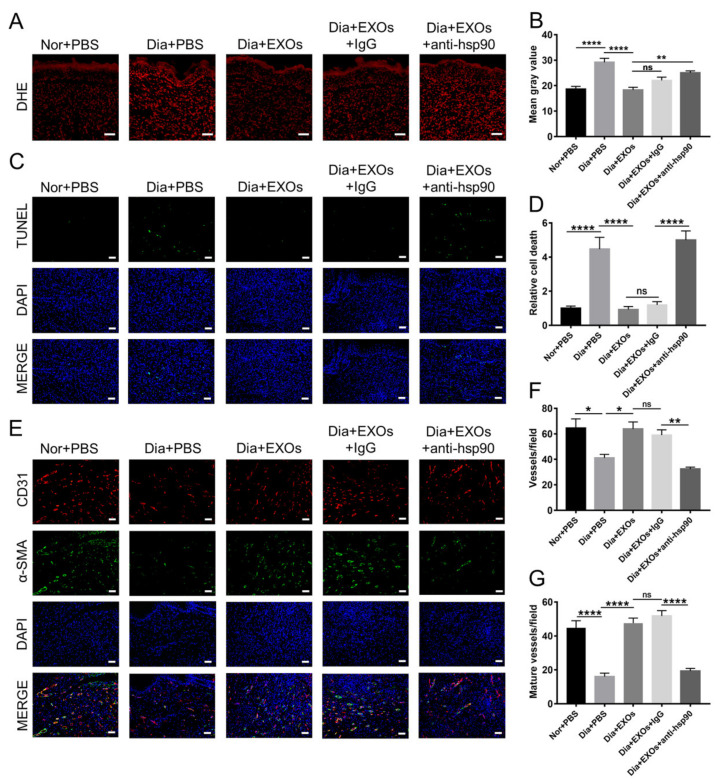
ADSC-EXOs enhanced neovascularization and reduced cell death in diabetic wounds via eHSP90. (**A**) Representative DHE fluorescent images of the wound section in each group; scale bar: 50 µm. (**B**) Quantitative analysis of the mean fluorescence intensity in (**A**). (**C**) TUNEL staining analysis of the apoptotic level of wound cells in each group; green color represents the dead cells, and blue color represents the cell nucleus; scale bar: 200 μm. (**D**) Quantification of the apoptotic rate shown in (**C**). (**E**) Immunofluorescence staining of CD31 (red) and α-SMA (green) in the wound sections on day 13 post operation; scale bar: 200 μm. (**F**) Enumeration of newly formed vessels stained with red color. (**G**) Enumeration of mature vessels co-stained with red and green colors. *n* = 7. * *p* < 0.05, ** *p* < 0.01, **** *p* < 0.0001.

Diabetic wounds contain a microenvironment characterized by hypoxia and high oxidative stress, which results in cell dysfunction and the potential for apoptosis. The ROS levels in wound sections were detected by dihydroethidium staining. The red fluorescence signals were stronger in diabetic wounds than in non-diabetic wounds, indicating that diabetic wounds had high ROS levels; moreover, ROS levels in diabetic wounds were dramatically diminished after treatment with ADSC-EXOs, but this effect was partially reversed by the use of anti-HSP90 antibody (Figure 7A,B). These data indicated that ADSC-EXOs exhibited excellent ROS-scavenging performance. Consistent with these findings, TUNEL staining showed that there were more apoptotic cells in diabetic wounds than in non-diabetic wounds; the number of apoptotic cells was significantly reduced after treatment with ADSC-EXOs in diabetic wounds. Furthermore, the use of an anti-HSP90 antibody was sufficient to antagonize the anti-apoptosis effect of ADSC-EXOs (Figure 7C,D). Taken together, the above results indicated that eHSP90 has a vital role in the attenuation of oxidative stress-induced cell apoptosis.

Immunofluorescence staining of CD31 and α-smooth muscle actin (α-SMA) on wound sections was performed to observe angiogenesis in vivo. The numbers of newly formed capillaries and new mature vessels were significantly higher in diabetic wounds treated with ADSC-EXOs than in untreated control wounds, while the use of an anti-HSP90 antibody reversed the effect of ADSC-EXOs; moreover, the neovascularization ability was weaker in diabetic wounds than in non-diabetic wounds (Figure 7E–G). In conclusion, diabetes obstructed wound neovascularization; eHSP90 efficiently promoted neovascularization in diabetic wounds.

We performed immunofluorescence staining of Ki67 and immunohistochemical staining of PCNA to evaluate cell proliferation in granulation tissue. The numbers of Ki67-positive cells and PCNA-positive cells were substantially increased after treatment with ADSC-EXOs in diabetic wounds; these effects were not reversed by the use of anti-HSP90 antibody (Figure S9). These results revealed that the mechanism by which ADSC-EXOs promote cell proliferation in diabetic wounds is independent of surface HSP90.

## 4. Discussion

**Figure 8:**
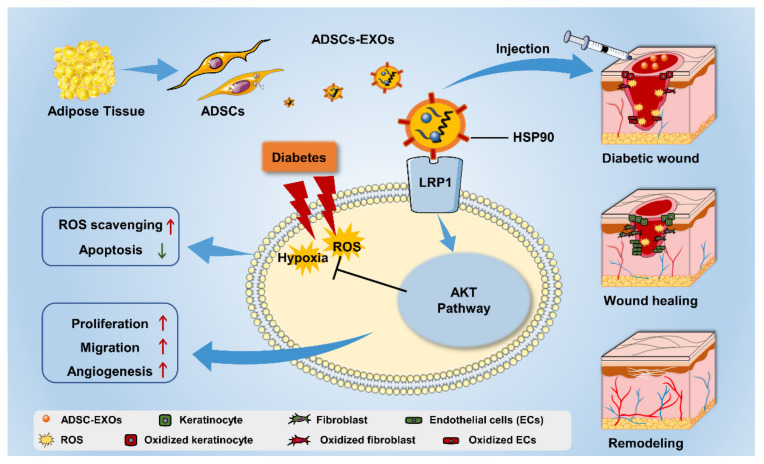
The mechanism of ADSC-EXO modulating diabetic wound-healing process.

Stem cell transplantation has been investigated in clinical trials as potential treatment for various diseases. Compared with cell therapy, exosome therapy has the following advantages: recapitulation of parental cell biological activity, efficient passage through biological barriers, low immunogenicity, and robust maneuverability [31,32]. There is increasing interest in the use of exosomes for wound-healing therapy, but few studies have focused on the regulatory effects of exosomes on oxidative stress in wounds. Here, we investigated the therapeutic effects of ADSC-EXOs on the modulation of oxidative stress in diabetic wounds along with the underlying mechanisms of such effects. In vitro and in vivo experiments revealed that ADSC-EXOs could protect against hypoxic and oxidative stress injury through surface HSP90, which targeted the LRP1 receptor and activated the AKT signaling pathway, thereby promoting diabetic wound healing (Figure 8).

Wound healing is a complex and well-orchestrated biological process; it is commonly divided into four overlapping and sequential phases (hemostasis, inflammation, proliferation, and remodeling) [33]. In the proliferation phase, keratinocytes participate in the construction of new skin epidermis; fibroblasts are an important component cell of formed granulation tissue; and endothelial cells are involved in the formation of new vascular networks [29]. Therefore, the promotion of cell function during this phase can accelerate wound healing. ADSCs constitute a subset of MSCs that reportedly release exosomes, which play important roles in wound repair by regulating inflammation, accelerating cell proliferation and migration, and promoting angiogenesis [34,35]. Here, we successfully extracted high-purity ADSC-EXOs by differential ultracentrifugation; we systematically evaluated the effects of ADSC-EXOs on three key cell types involved in wound healing. Consistent with the findings in previous reports, we showed that ADSC-EXOs could enhance proliferation and migration in HaCaT cells, fibroblasts, and HUVECs in vitro. Moreover, we found that ADSC-EXOs significantly increased the number of capillary-like tubular structures, implying that ADSC-EXOs could enhance angiogenesis. These findings suggest that ADSC-EXOs can promote the functions of three skin cells during wound healing.

Oxidative stress is involved in the occurrence and development of diabetic wound healing [5]. In general, the balance of ROS generation and elimination is precisely maintained by intracellular antioxidant protective systems. However, in a state of oxidative stress, excessive ROS leads to redox imbalance in the diabetic wound microenvironment; thus, the endogenous antioxidant system cannot completely prevent damage [36]. The use of exogenous antioxidants has become an effective method to eliminate oxidative stress damage [37]. Antioxidant enzymes (e.g., superoxide dismutase, catalase, glutathione peroxidase, and heme oxygenase), vitamins, medicinal plants, and nanoparticles are used as major antioxidants for wound repair [38]. Recent studies have shown that exosomes can regulate oxidative stress, but the underlying mechanism has been unclear. Li et al. demonstrated that ADSC-EXOs overexpressing Nrf2 can reduce ROS levels and inflammatory cytokine expression levels in endothelial cells exposed to high glucose, thereby promoting endothelial cell survival [39]. Human umbilical cord MSC-derived exosomes have been shown to reverse H_2_O_2_ and CCl_4_-induced oxidative stress damage and apoptosis in liver cells through the delivery of glutathione peroxidase 1 [40]. In the present study, we noted that ADSC-EXOs had a significant antioxidant effect in H_2_O_2_-stimulated cells. The ROS levels and cell apoptosis were increased in HaCaT cells, fibroblasts, and HUVECs after H_2_O_2_- or COCl_2_-induced injury; these effects were reversed by ADSC-EXO treatment. Mitochondrial dysfunction is a common feature of delayed wound healing, which causes excessive ROS generation [41]. Previous studies have demonstrated that ADSC-EXOs have beneficial effects on treating liver ischemia reperfusion injury and amyotrophic lateral sclerosis by regulating mitochondrial functions [42,43]. Whether ADSC-EXOs could reduce the intracellular ROS level of skin cells through rescuing mitochondrial functions needs be further studied.

Exosomes are cell–cell communication messengers that deliver their contents to recipient cells through three main pathways: ligand–receptor interaction, internalization, and direct membrane fusion [44]. Most exosome research focuses on internalization and direct membrane fusion. For example, exosomes from bone marrow MSCs were internalized by HUVECs [12]. In another study, exosomes from human induced pluripotent MSCs were taken up by HaCaT cells and human dermal fibroblasts [45]. However, in the present study, we detected minimal uptake of PKH26-labeled exosomes by fibroblasts despite incubation for 24 h, while those exosomes were rapidly internalized by 293T cells, HaCaT cells, and HUVECs. Subsequent co-culture experiments involving fibroblasts and Schwann cells confirmed our findings. In addition, we noted that HUVECs formed capillary tubes within 2 h of ADSC-EXO treatment; the AKT and ERK signaling pathways were activated with 30 min of ADSC-EXO treatment. Considering the rapid signaling response, we inferred that ADSC-EXOs might exert effects on wound cells partially through ligand-receptor interactions. Wound healing is a complicated biological process, which requires the accurate cooperation of many different biological and molecular events. Interestingly, the current data show numerous mechanisms are involved in the action of stem cell exosomes on wound healing, including the Notch, ERK, AKT, STAT-3, and Wnt/β-catenin signaling pathways [46]. These pathways may work together and exert multiple functions in different stages of wound healing. Therefore, the effect of ADSC-EXOs on other signaling pathways should be fully investigated in further studies.

Ligand–receptor interactions have regulatory roles that mainly involve exosomal membrane proteins, which bind to the recipient cell surface and initiate intracellular signaling pathways [47,48]. To determine which exosomal membrane proteins mediated antioxidant effects, we analyzed proteins present in ADSC-EXOs. Among the 10 most abundant proteins on the plasma membrane, HSP90 was selected for further analysis because it is involved in the wound-healing process. Previous studies have demonstrated that HSP90 is present on the surface of tumor-secreted exosomes and that anti-HSP90 antibody nullified the pro-motility activity of those exosomes [49]. Our results showed that an anti-HSP90 antibody could neutralize the effects of ADSC-EXOs in terms of promoting cell migration and protecting cells against hypoxic and oxidative stress damage. Therefore, we confirmed that ADSC-EXOs mediated their effects on fibroblasts through ligand-receptor interactions, using surface HSP90 as the effector molecule. HSP90 has been reported to bind to LRP1, human epidermal growth factor receptor 2, and TLR4 [24]. LRP1 is a ubiquitous cell-surface receptor that mediates hypoxia-induced cell migration, cancer cell survival, and epithelial-to-mesenchymal transition [24,50]. HSP90 binding to LRP1 has been shown to promote cell migration and survival during wound healing [21,50]. Here, we found that the positive effects of ADSC-EXOs in terms of promoting cell migration and survival were abrogated in LRP1 knockdown fibroblasts, confirming that LRP1 is the membrane receptor for ADSC-EXOs.

Extracellular HSP90 binding to LRP1 has been reported to activate multiple signaling pathways, including the AKT, ERK, and NF-κB signaling pathways [49,51]. We found that ADSC-EXOs could activate the ERK and AKT signaling pathways (but not the NF-κB signaling pathway) in fibroblasts; this activation could be inhibited by an anti-HSP90 antibody or by knockdown of LRP1. Furthermore, LY294002 effectively antagonized the effects of ADSC-EXOs in terms of promoting fibroblast migration and protecting fibroblasts against hypoxic and oxidative stress damage, whereas U0126 could not antagonize those effects. Collectively, these results showed that HSP90 on the exosomal surface activated the AKT signaling pathway by binding to the LRP1 receptor; it thus promoted cell migration and improved cell survival under oxidative stress.

Finally, we observed the effects of ADSC-EXOs on wound healing in established diabetic wound models. As expected, ADSC-EXOs significantly promoted collagen deposition and neovascularization, thereby facilitating diabetic wound closure; the addition of an anti-HSP90 antibody reversed the effects of ADSC-EXOs. The microenvironment of diabetic wounds is in a state of oxidative stress with high levels of ROS. Previous analyses of stem cells and exosomes have not extensively investigated ROS levels in wounds. We found that ADSC-EXOs exhibited excellent ROS-scavenging and anti-apoptosis performance although these effects were antagonized by the use of anti-HSP90 antibody. However, the anti-HSP90 antibody could not antagonize the effects of ADSC-EXOs in terms of promoting cell proliferation in diabetic wound models.

## 5. Conclusions

In this study, we demonstrated that ADSC-EXOs could promote skin cell proliferation, migration, and survival during exposure to hypoxic and oxidative stress damage; they could also facilitate collagen deposition and neovascularization, which led to accelerated diabetic wound healing. The underlying mechanism involved their surface eHSP90, which bound to the LRP1 receptor and activated the downstream AKT signaling pathway. Thus, our findings suggest that the application of ADSC-EXOs can enhance skin healing in diabetic wounds.

## Data Availability

Data presented in this study are contained within this article and in the supplementary materials, or are available upon request to the corresponding author.

## Supplementary Materials

The following supporting information can be downloaded at: https://www.mdpi.com/article/10.3390/cells11203229/s1, Figure S1: Identification of ADSCs. Figure S2: Identification of ADSCs-EXOs. Figure S3: ADSC-EXOs promoted skin cell proliferation. Figure S4: ADSC-EXOs promoted skin cell migration. Figure S5: ADSC-EXOs promoted endothelial cell angiogenesis. Figure S6: ADSC-EXOs protect skin cells against damage from hypoxia and oxidative stress. Figure S7: ADSC-EXOs activated the AKT and ERK signaling pathways in skin cells. Figure S8: U0126 (an ERK inhibitor) could not affect the function of ADSC-EXOs. Figure S9: ADSC-EXOs promoted cell proliferation in diabetic wounds; Table S1: The sequences of three different sh-LRP1. Table S2: Protein Report. Table S3: GO analysis of protein.

## Abbreviations

ADSCs	Adipose-derived mesenchymal stem cells
ADSC-EXOs	Exosomes from adipose-derived stem cells
ROS	Reactive oxygen species
HSP	Heat-shock protein
HSP90	Heat-shock protein 90
LRP1	Low-density lipoprotein receptor-related protein 1
eHSP90	Extracellular HSP90
TLR2 and TLR4	Toll-like receptors 2 and 4
DCFH-DA	2,7-dichlorofluorescein diacetate
PCNA	Proliferating cell nuclear antigen
