# Supplementary material for: Exosomes from Adipose Stem Cells Promote Diabetic Wound Healing through the eHSP90/LRP1/AKT Axis
Source: Cells. 2022 Oct 14;11(20):3229. doi: 10.3390/cells11203229 (PMC9600018; doi:10.3390/cells11203229)
Supplement: Supplementary file 1 [file cells-11-03229-s001.zip › Supplementary information 0918.pdf]

Exosomes from adipose stem cells promote diabetic wound healing through the eHSP90/LRP1/AKT axis

Sen Ren[1,2, #], Jing Chen[1, #], Jiahe Guo[1, #], Yutian Liu[1], Hewei Xiong[3], Boping Jing[4], Xiaofan Yang[1], Gongchi Li[1], Yu Kang[1], Cheng Wang[1], Xiang Xu[1], Zhenyu Liu[1], Maojie Zhang[1], Kaituo Xiang[1], Chengcheng Li[1], Qianyun Li[1], Hans-Günther Machens[5], Zhenbing Chen[1, *]

[1]Department of Hand Surgery, Union Hospital, Tongji Medical College, Huazhong University of Science and Technology, Wuhan 430022, China

[2]Department of Neurosurgery, Zhongnan Hospital of Wuhan University, Wuhan 430071, China

[3]Department of Emergency Surgery, Union Hospital, Tongji Medical College, Huazhong University of Science and Technology, Wuhan 430022, China

[4]Department of Ultrasound, Union Hospital, Tongji Medical College, Huazhong University of Science and Technology, Wuhan 430022, China

[5]Department of Plastic and Hand Surgery, Technical University of Munich, Munich, Germany.

[#] These authors contributed equally to this work.

[*]Corresponding author: Zhenbing Chen; E-mail: zbchen@hust.edu.cn.

Department of Hand Surgery, Union Hospital, Tongji Medical College, Huazhong University of Science and Technology, No. 1277 Jiefang Avenue, Wuhan 430022, China. Tel: +86 13871103730.

Figure S1

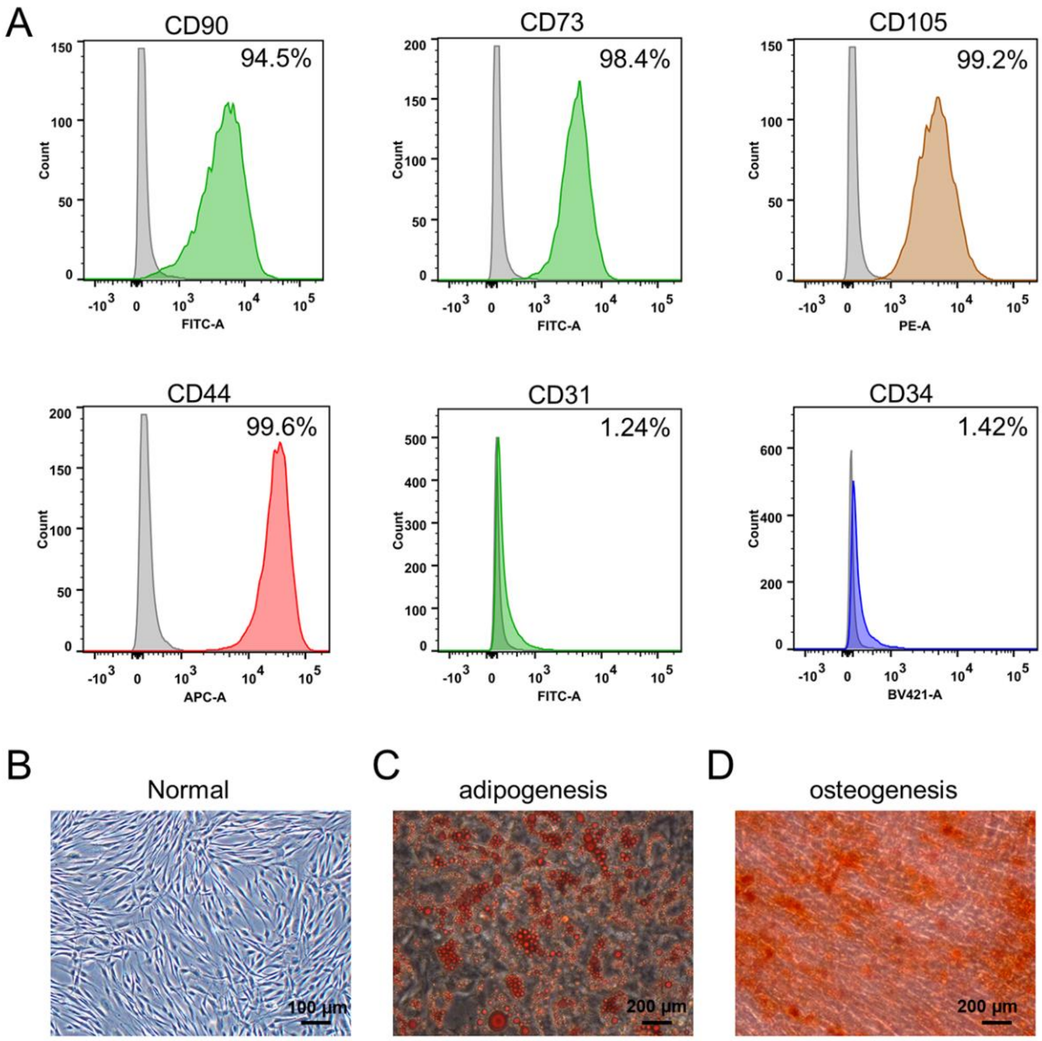

Figure S1: Identification of ADSCs. (A) Flow cytometry analysis of ADSCs' surface markers including CD90, CD73, CD105, CD44, CD31, and CD34. (B) Representative images of normal cultured ADSCs under a microscope; scale bar: 100 µm. (C) Representative images of ADSCs after adipogenic differentiation for seven days; scale bar: 200 µm. (D) Representative images of ADSCs after osteogenic differentiation for three weeks; scale bar: 200 µm.

Figure S2

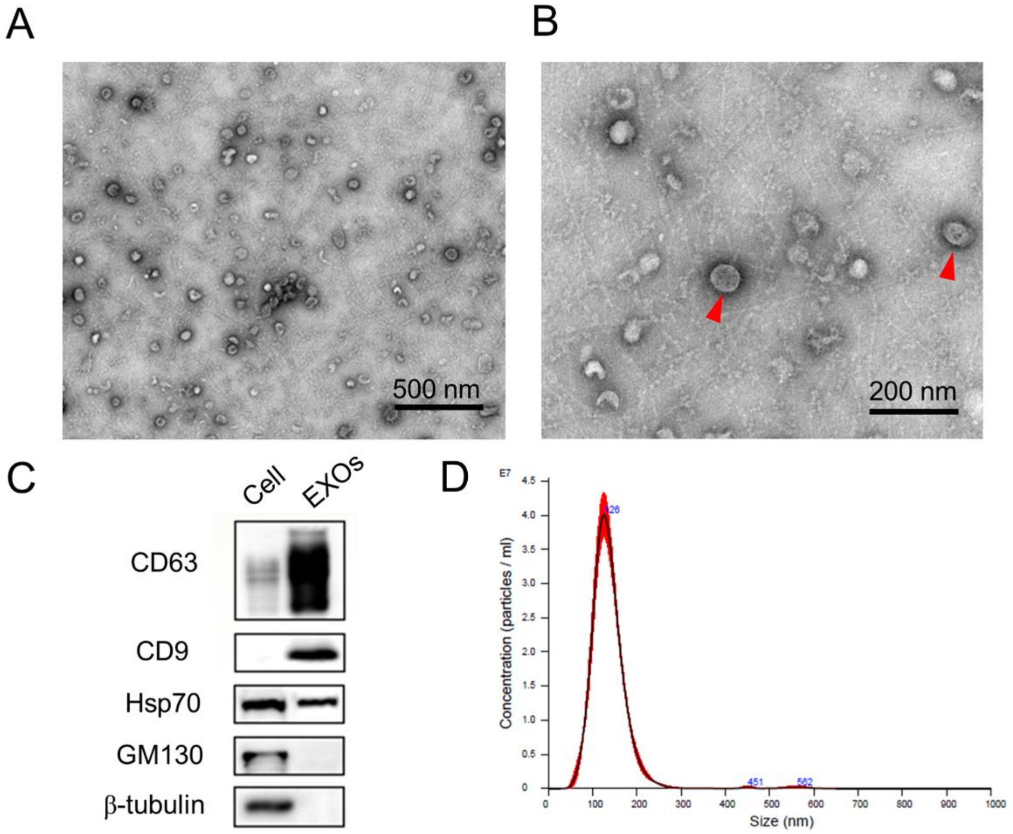

Figure S2: Identification of ADSCs-EXOs. (A, B) Transmission electron microscope images of ADSC-EXOs morphology. (C) Western blot analysis of ADSC-EXOs markers including CD63, CD9, HSP70, GM130 and β-tubulin. (D) Nanoparticle tracking analysis of isolated ADSC-EXOs.

Figure S3

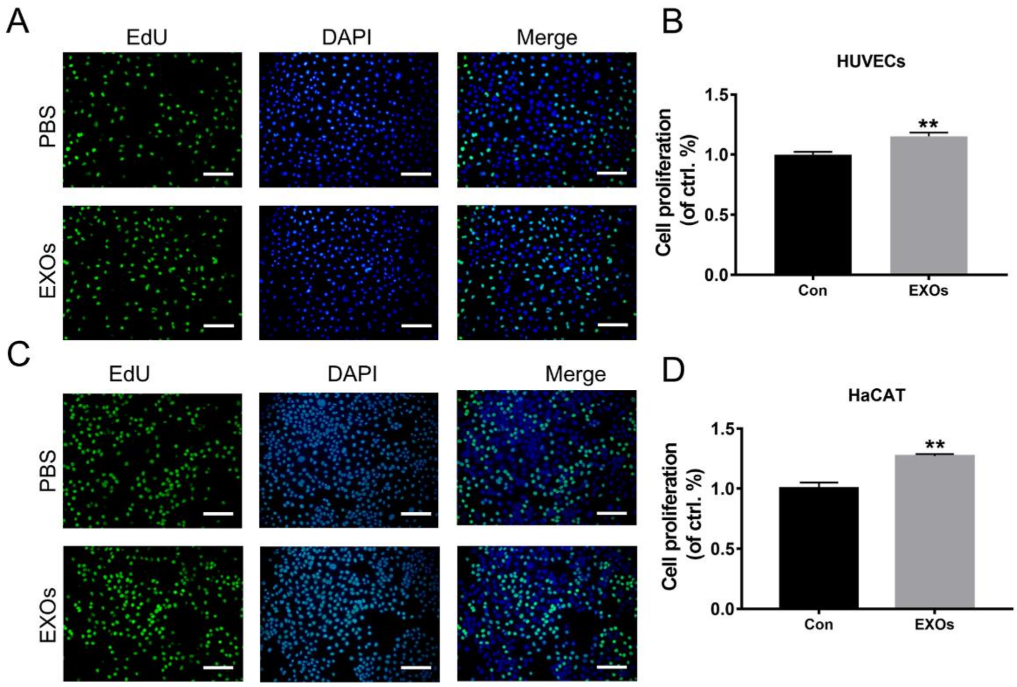

Figure S3: ADSC-EXOs promoted skin cell proliferation. Skin cells were treated with ADSC-EXOs or an equal volume of PBS for 24 h, and then the cellular proliferative rate was detected by EdU assay; the green color represents proliferative cells, and the blue color represents the cell nucleus. (A) Representative fluorescent images of proliferative HUVECs after ADSC-EXOs treatment. (B) Quantitative analysis of the data in A. (C) Representative fluorescent images of proliferative HaCaT after ADSC-EXOs treatment. (D) Quantitative analysis of the data in C. $N = 5$. Scale bar: 100 μm. $^{**}p < 0.01$.

Figure S4

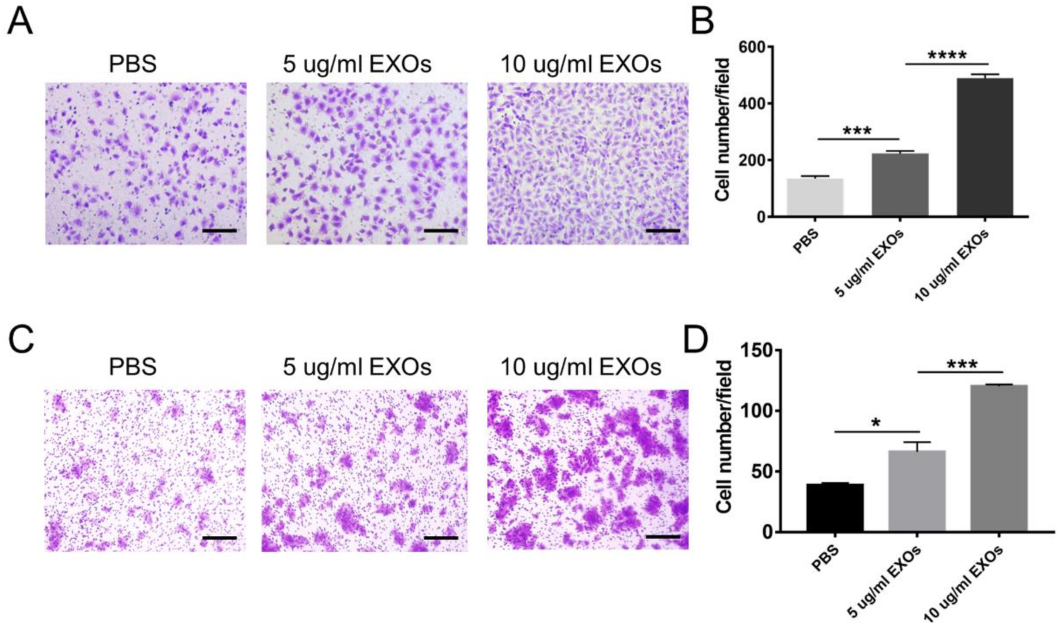

Figure S4: ADSC-EXOs promoted skin cell migration. Skin cells were treated with ADSC-EXOs (5 or 10 μg/ml) or an equal volume of PBS for 24 h, and then migratory cells were detected by Transwell assay. (A) Representative images of migratory HUVECs given above treatments; scale bar: 100 μm. (B) Quantitative analysis of the data in A. (C) Representative images of migratory HaCaT given above treatments; scale bar: 200 μm. (D) Quantitative analysis of the data in C. *N* = 5. *p< 0.05, **p< 0.01, ***p< 0.001, ****p< 0.0001.

Figure S5

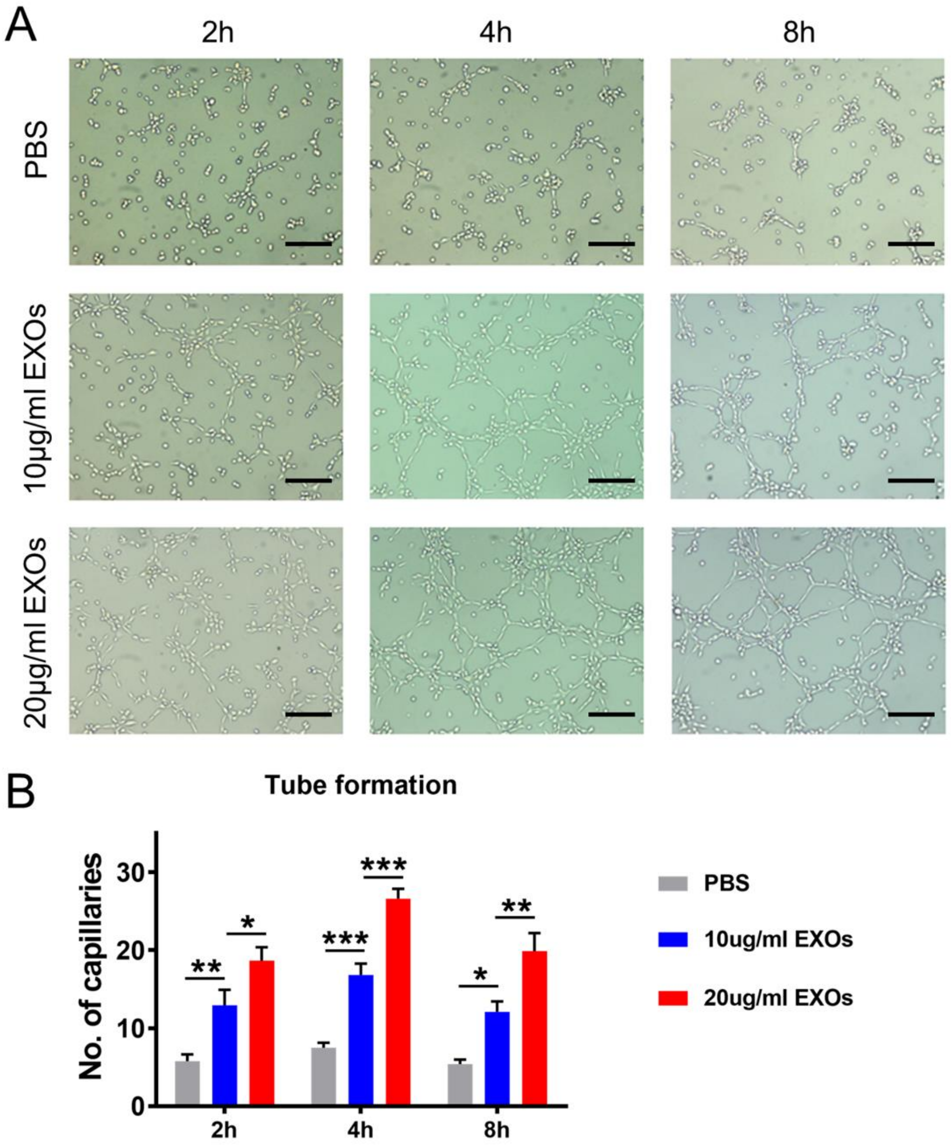

Figure S5: ADSC-EXOs promoted endothelial cell angiogenesis. HUVECs were treated with ADSC-EXOs (10 or 20 μg/ml) or an equal volume of PBS, and then seeded in 48-well plates precoated with Matrigel Basement Membrane Matrix. (A) Representative images of tube formation in HUVECs given above treatment at different time points. (B) Quantitative analysis of the data in A. *N* = 3. Scale bar: 200 μm. *p< 0.05, **p< 0.01, ***p< 0.001.

Figure S6

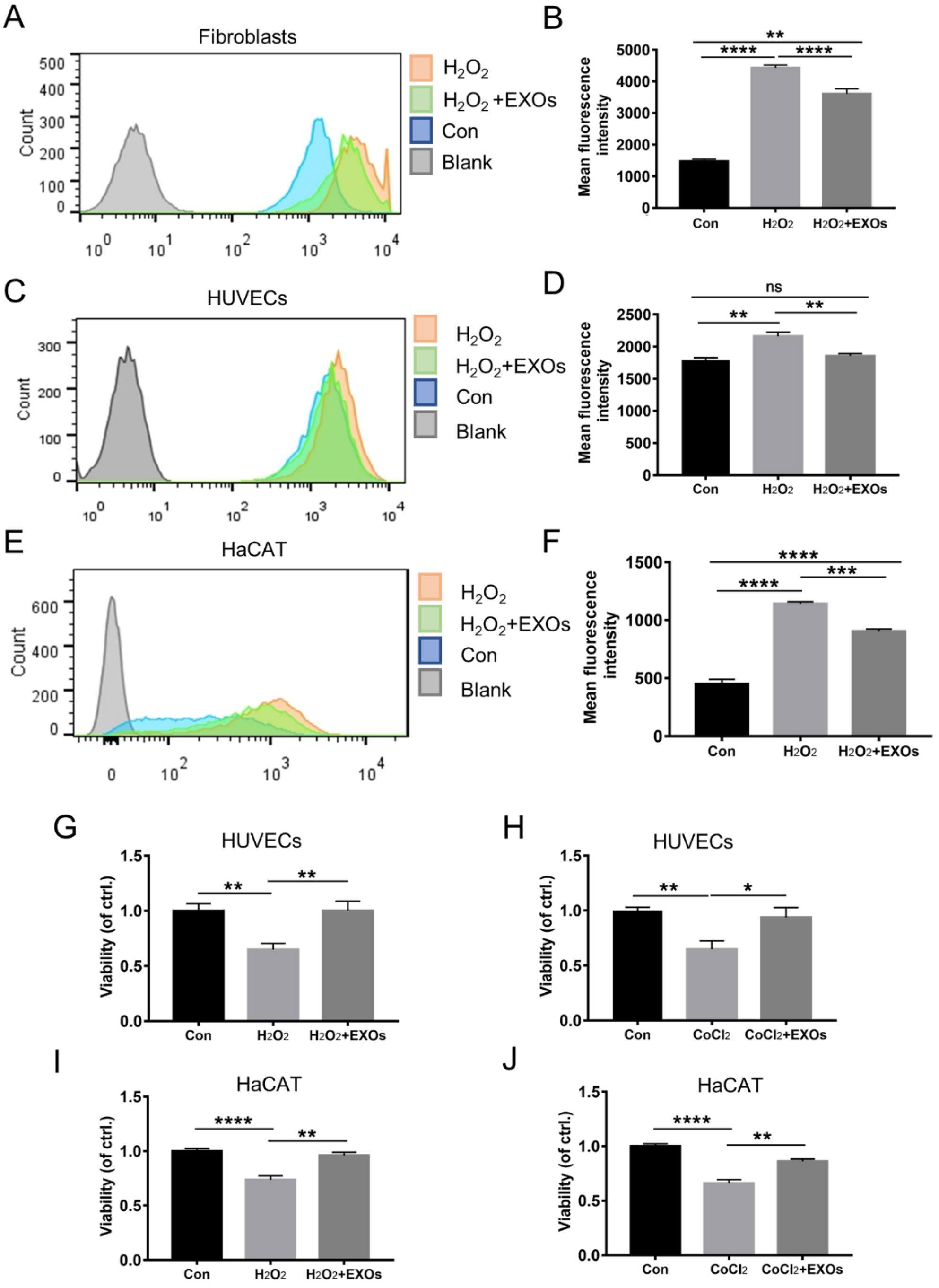

Figure S6: ADSC-EXOs protect skin cells against damage from hypoxia and oxidative stress. Skin cells were pre-treated with ADSC-EXOs or PBS for 24 h, then exposed under H_2O_2 for 6 h or $CoCl_2$ for 24 h, and finally assessed by the following assays. (A) Flow cytometry analysis of the ROS levels in fibroblasts given above treatments. (B) Quantitative analysis of the mean fluorescence intensity shown in A. (C) Flow cytometry analysis of the ROS levels in HUVECs given above treatments. (D)

Quantitative analysis of the mean fluorescence intensity shown in C. (E) Flow cytometry analysis of the ROS levels in HaCaT given above treatments. (F) Quantitative analysis of the mean fluorescence intensity shown in E. (G, H) Quantitative analysis of the cell viability of HUVECs under H_2O_2 or $CoCl_2$ exposure by the CCK8 assay. (I, J) Quantitative analysis of the cell viability of HaCaT under H_2O_2 or $CoCl_2$ exposure by the CCK8 assay. $N = 5$. *p< 0.05, **p< 0.01, ***p< 0.001, ****p< 0.0001.

Figure S7

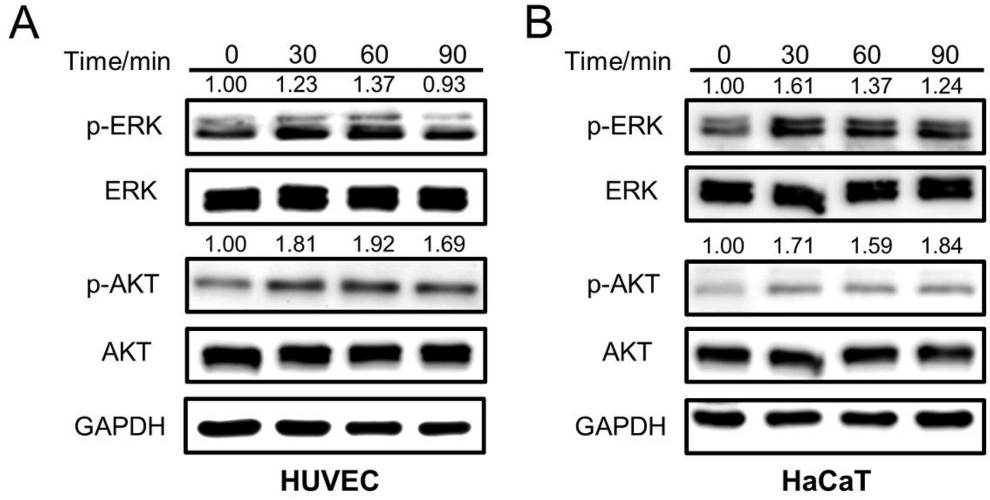

Figure S7: ADSC-EXOs activated the AKT and ERK signaling pathways in skin cells. (A) Western blot analysis of the activation of AKT and ERK pathways in HUVEC after treated with ADSC-EXOs at different times (n=3). (B) Western blot analysis of the activation of above pathways in HaCaT after treated with ADSC-EXOs at different times (n=3).

Figure S8

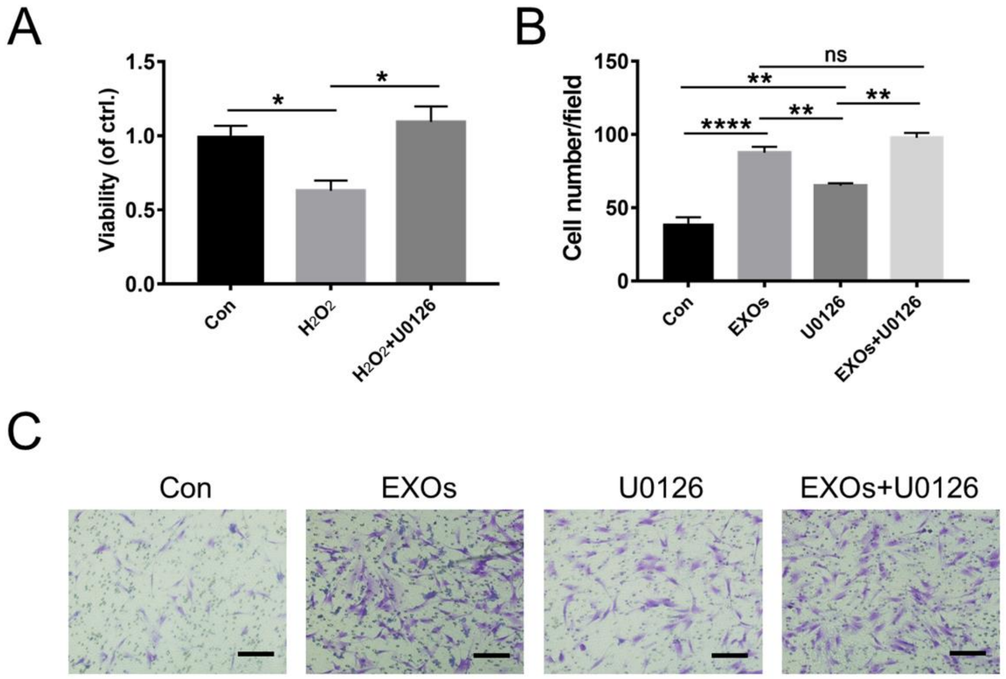

Figure S8: U0126 (an ERK inhibitor) could not affect the function of ADSC-EXOs. (A) CCK8 analysis of the cell viability of fibroblasts given EXOs, EXOs + U0126, or PBS treatments for 6 h. (B) Quantitative analysis of the data in C. (C) Representative micrographs of migratory fibroblasts given EXOs, EXOs + U0126, U0126, or PBS treatments for 24 h. *N* = 5. Scale bar: 100 μm. *p< 0.05, **p< 0.01, ***p< 0.001, ****p< 0.0001.

Figure S9

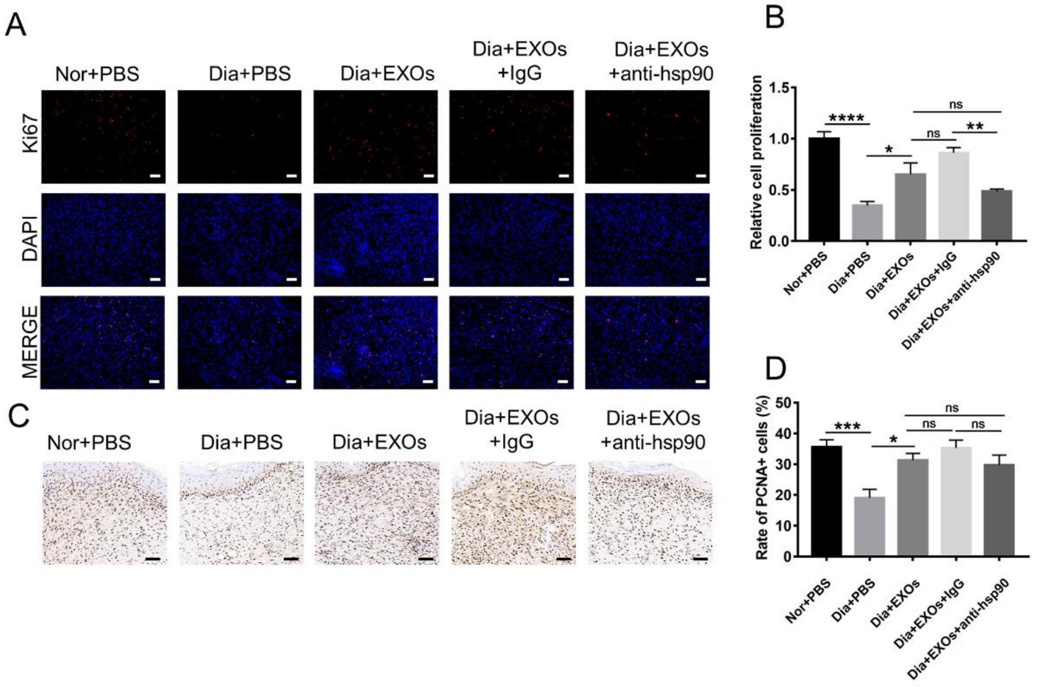

Figure S9: ADSC-EXOs promoted cell proliferation in diabetic wounds. (A) Immunofluorescence staining of Ki67 (red) in the wound sections on day 13 post-operation; scale bar: 200 μm. (B) Quantification of the relative proliferative rate shown in A. (C) Immunohistochemical staining of PCNA in the wound sections on day 13 post-operation; scale bar: 50 μm. (D) Quantification of the proliferative rate shown in C. $N = 7$. *p< 0.05, **p< 0.01, ***p< 0.001, ****p< 0.0001.

Table S1

NO.	5’	STEM	Loop	STEM	3’
Sh-LRP1-#1-a	Ccgg	gcGAACAAACAC ACTGGCTAA	CTC GAG	TTAGCCAGTGT GTTTGTTCGC	TTTTT g
Sh-LRP1-#1-b	aattca aaaa	gcGAACAAACAC ACTGGCTAA	CTC GAG	TTAGCCAGTGT GTTTGTTCGC	
Sh-LRP1-#2-a	Ccgg	ccTGCAACAATGG CAGATGTA	CTC GAG	TACATCTGCCA TTGTTGCAGG	TTTTT g
Sh-LRP1-#2-b	aattca aaaa	ccTGCAACAATGG CAGATGTA	CTC GAG	TACATCTGCCA TTGTTGCAGG	
Sh-LRP1-#3-a	Ccgg	GACCAGTGCTCT CTGAATA	CTC GAG	TATTCAGAGAG CACTGGTC	TTTTT g
Sh-LRP1-#3-b	aattca aaaa	GACCAGTGCTCT CTGAATA	CTC GAG	TATTCAGAGAG CACTGGTC	

Table S1: The sequences of three different sh-LRP1.
